# Diet in Disease

**Published:** 1892-10-08

**Authors:** Ernest Hart

**Affiliations:** Bachelier-es-Sciences-es-Lettres (restreint), Medical Student of the Faculty of Medicine of Paris, and of the London School of Medicine for Women


### Diet in Disease.

By Mrs. Ernest Hart, Bachelier-es-Sciences-es-Lettres (restreint), Medical Student of the Faculty of Medicine of Paris, and of the London School of Medicine for Women.

### II.—FOOD AND FOOD VALUES (*continued*).

### Hydro-Carbons and Carbo-Hydrates.

*Fats. Sugar. Starch.*

In the group of Hydro-Carbons or Fats are included all vegetable and animal fatty foods, such as oils, suet butter, &c.

The value and necessity of fat as an article of food is obvious from the fact that the outer covering of the body beneath the skin is composed of fat. It is fat or adipose tissue which gives the rounded form and curved lines that constitute, according to our ideas, one of the essentials of beauty, and it is moreover this outer covering of fat which protects the body and organs from sudden changes of temperature.

Fat is in constitution a much simpler food principle than the albuminates, and it is shown by analysis to be composed as follows:—

| | |
|---|---|
| Carbon | 79 |
| Hydrogen | 11 |
| Oxygen | 10 |
| | 100 |

*Uses of Fat.*—It was taught by Liebig that fat split up in the body, and that the freed carbon combined with the oxygen taken in in respiration to produce carbonic acid ($CO_2$), and that it was by this act of respiratory combustion that the body heat was maintained. Fatty foods were hence considered necessary as heat-producers. Recent investigations have, however, proved that the matter is not so simple, and that though fat is split up and combined with oxygen in the production of heat, especally during muscular exercise, the process is effected in the tissues by the action of the cells, and not in the lungs; as formerly taught. The use of fat seems to be threefold. (1) To maintain the body heat. In cold latitudes, where the body is subject to rapid cooling, fatty foods become a necessity, so that the carbon may be easily supplied for combination with oxygen in combustion. Hence the Greenlander consumes large quantities of blubber and oil. (2) To produce force. As muscular force is only produced at the cost of oxidation in the tissues, fat is rapidly burned off during exercise. If absent, the tissues themselves would be wasted, and this fact brings us to the third use of fat, viz., to prevent the waste of albumen. If albuminous food alone be given a very large amount must be consumed for the body to obtain the elements necessary for the production of heat and mechanical energy; but if fat be added a much smaller amount of albumen is required. Hence there is no diet so wasteful as a purely albuminous diet, and it has been conclusively proved experimentally that a small amount of meat food taken in combination with bread and fat, suffices to maintain the albuminous structures of the body better than an exclusively lean meat diet.

Fat stored in the body as adipose tissue is a bank on which the body may draw for supplies of energy and heat when required. During great muscular exercise fat is rapidly decomposed and oxidised, and in the case of enforced starvation the body finds in adipose tissue the materials for its own support. It is stated that in the Franco-German War of 1870, the German Emperor, acting on the strongly expressed opinion of Ebstein that muscular fatigue could best be supported on fat, gave orders that each soldier should have served out to him 250 grammes of fat bacon. It is also a well-known fact that fat animals bear privation of food better than thin ones. Hybernating animals during their winter sleep live on the fat stored in their tissues, and awake in spring almost devoid of fat.

*Carbo-Hydrates* are composed of carbon, hydrogen, and oxygen; the hydrogen and oxygen always existing (in combination with the carbon) in such proportions as to form water. The chemical formula of water is $H_2O$; the chemical formula of starch is $C_6 H_{10} O_5$; from which it will be seen that starch is composed of six atoms of carbon and five molecules of water. The various substances in the group of carbo-hydrates, such as starch, dextrin, and sugar, are easily convertible one into the other by the action of ferments. The principal carbo-hydrates used in the food of man are starch and the various kinds of sugar. Starch is contained in all farinaceous foods and in many vegetables, as peas, beans, and potatoes. Sugar is found in the form of cane-sugar in the sugar-cane and beetroot, as milk sugar or lactose in milk, and as grape sugar or glucose in fruits. Considering, therefore, how largely our ordinary food consists of bread, potatoes, milk, and sugar, it will be seen that carbo-hydrates form very important elements in diet.

It is taught generally that the action of carbo-hydrates is that of *power producers* in the body; that is to say, that during muscular contraction, or, in fact, in order that it may take place, a carbo-hydrate must be split up, and its element of carbon combined with oxygen to produce carbonic acid, ($CO_2$). A muscle may be compared to a steam engine. The muscle fibres, composed of an albuminous material, correspond to the brass and steel of the engine. In order that the steam-engine may work, coal has to be burnt to produce power; and in the same way, in order for a muscle to work, carbo-hydrates have to be burnt to produce energy. Owing to constant use some of the metal parts of the machine wear away, so do some of the constituent parts of the muscle, and the refuse is thrown into the blood in the form of urea. In the engine, the rapid firing necessary for working at high pressure does not wear away the machine, but the consumption of coal must be increased and the production of carbonic acid much augmented, in the production of increased power. This is exactly what takes place during severe muscular exercise. The albuminous constituents of the muscles are not worn away, but the muscle requires the fuel of carbo-hydrates or of hydro-carbons in order that the carbon may be oxidised in the production of mechanical energy. This rapid oxidation is the cause of the increased heat of the body during muscular exertion. The carbonic acid produced is excreted by the lungs. In the experiments made by Dr. Edward Smith on himself it was shown that five times as much carbonic acid are exhaled when walking at the rate of three miles an hour, as during sleep, and twice this amount while working the treadmill, in which enforced exercise all the muscles of the body are continuously at work. From experiments made by various scientific observers in the ascent of mountains on a strictly non-nitrogenous diet, it has been ascertained that severe muscular exercise and fatigue can be well performed on a diet of carbo-hydrates only; in fact, it is now well established that the energy or power developed by muscular work in the body is produced by the oxidation or burning off of carbonaceous matter. Hence it results that fatty and starchy foods form an excellent dietary on which to do hard physical work.

In order that carbo-hydrates may be digested and assimilated, it is necessary that they should be converted into glucose. This is brought about by the action of the saliva and of the pancreatic juice, and will be described when digestion is treated of. The carbo-hydrates do not act only as energy-producers in the body; they also contribute to the formation of adipose tissue.

The *Production of Fat from Starchy and Sweet Foods* has been very much discussed, and a great number of experiments have been undertaken on living animals to determine whether this transformation takes place or not. The experiments by Huber, Grundlach, Dumas, and Milne-Edwards have proved that bees can manufacture the wax of the comb out of a diet of pure sugar or honey. From the observations of Persoz, a Professor of the Faculty of Science of Strasburg, on the production of *foie gras*, or fatty liver, in the Strasburg geese dedicated to the production of this pathological dainty, he found that far more fat was manufactured in the bodies of the geese than could be accounted for by the oily matter of the maize on which they were fed. The following record of an experiment by Tscherwinsky on pigs is also very conclusive. He took two pigs; No. 1 weighed 7,300 grammes, No. 2, 7,290 grammes. No. 1 was killed, and the fat and the albuminous constituents of its body were carefully weighed. No. 2 was kept four months and fed on an exclusive diet of grain. The animal was then killed. The amount of grain it had taken and the amount of albumen contained in the grain were carefully estimated. Its excreta were also analysed. After the pig was killed the amount of fat and albuminous substances contained in the body were estimated. The amount of fat that had been produced by the diet of grain could be ascertained on comparing pig No. 1 with pig No. 2. The following table gives the result:—

| | | | | | | |
|---|---|---|---|---|---|---|
| Pig No. 2 contained | 2·50 | kilos of | albumen and | 9·25 | kilos of | fat. |
| Pig No. 1 „ | 0·96 | „ | „ | 0·69 | „ | „ |
| Assimilated | 1·56 | „ | „ | 8·56 | „ | „ |
| Taken in in food | 7·49 | „ | „ | 0·66 | „ | „ |
| Difference | 5·93 | „ | „ | 7·90 | „ | „ |

It will thus be seen that only a small part of the albumen of the food was assimilated, or incorporated in the structure of the animal, but yet there was an increase of nearly 8 kilos of fat. Whence was this fat derived? The albumen which was not assimilated could, by splitting up, only furnish a very small part of it; and therefore the conclusion is inevitable that it was produced from the starch of the grain. How fat is produced from starch and sugar in the body is at present entirely unknown. The body is a laboratory of far greater complexity than the most perfectly fitted laboratory of the man of science, and the cells of the liver, the pancreas, and the muscles exercise wonderful powers in splitting up complex bodies into their component elements, and recombining these elements into new substances. After the most careful experiments have been made on the living body, the actual processes of life escape our detection and investigation; hence we do not actually know, we can only guess at the truth, and argue from the results of broad and coarse experiments.

Though it is now ascertained beyond dispute that fat is produced from carbo-hydrates, it has been proved by experiment that the production of fat is much more rapidly and easily effected if the carbo-hydrates are not given alone, but in conjunction with a small amount of fat. Thus Boussingault found that if pigs were fed exclusively on potatoes they would not fatten beyond a certain point; but if fed on potatoes mixed with wash, which contained a quantity of nitrogenous and fatty refuse thrown out of the kitchen, they fattened freely, and the fat which was produced in their bodies was ascertained to be greatly in excess of that given with the food, whence it was concluded that the small amount of fat and albumen ingested enabled the body to turn the starch of the potatoes into fat with greater ease.

*Cellulose*, which is the basis of construction of all plants, is a carbo-hydrate which the human sytem is incapable of digesting; it forms, however, the bulk of the food of the herbivorous animals in whose digestive system special arrangements are made for its digestion and assimilation. It is a matter of current observation that cows and horses will fatten on grass and hay, and their bodies must therefore have the power of converting the carbo-hydrate cellulose into fat. If, however, it is intended to produce an excessive deposit of fat, cattle are fed upon maize or oil-cake, in which the presence of oily substances contributes to the increased production of fat from the other constituents of the food.

